# Record of Meteorological Conditions at Buffalo, for May, 1855

**Published:** 1855-07

**Authors:** Sanford B. Hunt


					﻿ART. VI. — Record of Meteorological Conditions at Buffalo, for May,
1855. By Sanford B. Hunt, M. D.
2	~ fc Op,
<;	a	®	S
S	03	©"H	g
M	4>	2	2
rt	u	'g	g
aj 4> y >-»
_____________________________________________________________________________
a.tn;Bjadiusj, I
JO OJilHM jsajvojg|
————- —
-ptUItlH Uti.lft
5	•?«!<><!
MO(j tree ft
o —____________________________________________________________________________
I	I
-jadinaj, uBdj^l
„	CO	H	r-1 G$ CO G'J r-1 G^ I
• • vjipiuian	o -« io	oi cn gq Tt< b-
Z	1	co	bbbbxt'- o [
O "	--------------------------------------------------------------------
2
o •'lutoj Man	r“j *"1	u^.	l°.
fa. *	Qi	I G up (M	O X X X CS 0 I-
U £ .___________________________.___________1Q 1Q _________1Q CO CO CO UP 1Q
®	zT
n- •ain^duiaj,	.	...	................
Ph	b- T*bH	O GO CO CO O r-H
___________________________,	____________CkOi.G_________UP0	____
.	£	£	£
O £ § o ° £	W	TO	TO	TO	TO
Sj ____________________________________________O ci O -< O -' C O -< Q CO
g spno[Ojoj,uiy|	<=> oo o o ©©©©©© in j
p?	© <^ © d> o uo oo -i cw ® ci h r-i f t' - oo ip Tf o <c - « t' -~Td
*	•firnTrnnTT	O O M N « C-Ifi -	00-< » CP » O O O’d'O U, CO O lC «t—
«	A)!P!UItlH ao co co c— t- i.c I- ip X1 h r- I- i'i' D o o co i- io ‘O t'i/: lo lo lo rf io t' co
§ a -------------------——---------------------------------------------------------
8	-iuio-t Mon	“g ~“.	. in	©	©	c-. d c m « o io o i.o	o	h	io	-1 ?! . t-	oo
p	. <i u	, d ^ © -t<	i^ —'	i-'	©	© o c1 d' ©' -c* © © ©' © ci ci	©'	cd	—i	©	r-4 o ci ©'	c3
, •	h.	_©	© © © -f	31 W	W	Tl<	Tf 1." T i.1 .3 ifi ©©©©©© I-O	-t	C	-i1	-1	io io 'CO	©
s 5	--------------------------------------------------- I
r,	’*	«ONCToa>mmocooooo^’#o<-it'ioO'#occ>c>(N ct
“■< ________ © © © © ©	© -r © © © © © © © © ©	© © © t-^	© © © © t© t- t— t~- 'co
S^^'2	.M
r= § 2 c £	TO TO TO TO K TO TO TO TO TO !?J TO TO
o ?	...	......
_____________________________________________________________Ci^-Hr-HCicicicQr-HrH-^CQ^GQOUrH—<co
stfnQOJo^aiy______________________O.od coo* upoopooog* * * o
0b00G^^0-^’Hr-<C^’7<’^0LOCOCQCO
. •Xiroiinnu	co gq co up —< □ o x h G* r-co c*: g x cc >0
u	Cl GO 0 b 1-0 X0 bbXC'-b-bt- i> b—b”
o __________________________________________________________________________
a>	~	——
8	.1nr„T ua„	Cl	Cl Cl © d © © © 00 © OlOl^ t- I- i©
©	P	4 • d ■"■<11	t't3 I'-' Uo fi’f C£ C C O U IO o' -h
•=<	So	___________________ ©	©©©©©©©~J ©©©©©©©©©
J	■
- a-in^daiaj,	d^'ai^ 0 -*6 hig 6 lg oo ?: x 0 b
b-	_______________upupupUPUpCOUpUpUPCOUpUPUPiQ^UpCgb^
■< S t> 2. "3	^' •	• !>■ S.’
o 5 g = £	TO © fe^^TO TO TO TO	TO
# .... ......................
_______________________r-JrUoO^C^(^r-4oOGQ^^r-<r-MOOCO
SpnoIO jo^my	2 ogoo ©® ° o o « ooco oou>£
~ " ' 1---------------------------«
d	ci	ci . ©	.	ff? . - A
f- 'UI0'reS^0-nI ci cj ci	ci S ci	ci ci ci ci	ci ci ci ci C>	ci ci ci ci ci ci ci ci cn	00 ©
-	I CT CT CT CT CT_CT CT CT « CT CT Cl d d Cl CT CT CT CT CT CT Cl C1 Cl Cl Ct
<	° ®	i2
3 •uti>HJ°’uI	© r~	o
~	Wi-|CTW'*ia(Otw|10a,SJ2 23!22^22gCTCTCTCTCTCTCTCTCT°J~
"Hazy.
				

